# Temporal sequencing of sensory impairments and frailty reveals vulnerability-dependent dementia risk

**DOI:** 10.21203/rs.3.rs-10318713/v1

**Published:** 2026-07-16

**Authors:** Shichen Zhou, Markus J. Haapanen, Emily H. Gordon, Kenneth Rockwood, Ruth E. Hubbard, Piers Dawes, David D. Ward

**Affiliations:** 1 Frazer Institute, Faculty of Health, Medicine and Behavioural Sciences, The University of Queensland, Woolloongabba, Australia; 2 Australian Frailty Network, The University of Queensland, Woolloongabba, Australia; 3 Research Unit of Clinical Medicine, University of Oulu, Oulu, Finland; 4 Folkhälsan Research Centre, Helsinki, Finland; 5 Division of Geriatric Medicine, Department of Medicine, Dalhousie University, Halifax, Nova Scotia, Canada; 6 Geriatric Medicine & Neurology, Nova Scotia Health, Halifax, Nova Scotia, Canada; 7 School of Health and Rehabilitation Sciences, Faculty of Health, Medicine and Behavioural Sciences, St Lucia, The University of Queensland, Australia; 8 Geriatric Medicine, Princess Alexandra Hospital, Metro South Hospital and Health Service, Woolloongabba, QLD, Australia

## Abstract

Although hearing and vision impairments are considered modifiable dementia risk factors, it remains unclear whether their associations reflect sensory-specific pathways or broader ageing-related vulnerability. Using harmonised data from the Health and Retirement Study (n=22,756) and the English Longitudinal Study of Ageing (n=12,921), we applied multi-state Markov models, time-varying Cox models and disaggregated population attributable fractions (PAFs) to test whether associations depend on their temporal sequence with frailty. Associations weakened after frailty adjustment (adjusted hazard ratios, aHRs, 1.17–1.80 [PAFs 3.4–9.2%] vs 1.08–1.56 [PAFs 1.5–6.6%]). Sensory impairments arising before frailty showed no independent association with incident dementia (aHRs 0.88–1.22 [PAFs −1.3 to 1.5%]), whereas those emerging with or after frailty were consistently associated (aHRs 1.31–2.28 [PAFs 3.4–6.4%]). Conventional estimates may over-attribute dementia burden to sensory impairments by neglecting their ageing context. Frailty-informed approaches may improve targeting and interpretation of sensory interventions for dementia risk reduction.

More than 57 million people worldwide have dementia, with cases projected to almost triple by 2050^1^. Despite an intense focus on single protein abnormalities, most dementia develops in later life. Late-life dementia is characterised by mixed neurodegenerative, vascular and systemic risks accumulated over decades^2,3,4^, complicating the development of disease-modifying therapeutics^5^. These challenges highlight the importance of developing effective, equitable and scalable public health interventions to reduce risk of dementia^6^. Sensory impairments—hearing and vision—are of particular interest because they are common, are often easily detected and at least partially corrected, and are consistently associated with higher rates of incident dementia and cognitive decline in observational studies^7,8,9,10,11,12,13,14^. Proposed mechanisms include increased cognitive load, social and physical disengagement, accelerated regional brain atrophy, and shared microvascular and neurodegenerative substrates^2,15,16^. Previous analyses suggest that, in combination, hearing impairment and vision impairment may account for almost 10% of dementia cases (approximately 7% and 2% respectively)^17,18^. However, with ageing, sensory impairments accrue frequently alongside frailty^19^, raising uncertainty about whether their associations with dementia development reflect potentially modifiable sensory impairment, broader ageing-related vulnerability, or both.

Ageing is more than the passing of time and reflects cumulative physiological, functional, and cognitive change, rates of which vary considerably among individuals of the same chronological age^20,21^. Frailty—a measurable health state of vulnerability to adverse outcomes^22^—captures heterogeneity in ageing, providing an estimate of biological age that enables risk stratification of individuals of the same chronological age^23^. Operationalised under the deficit-accumulation model that integrates multidomain deficits—clinical, functional, and behavioural—frailty index scores closely track mortality across species even after adjustment for chronological age^24,25^. Across populations and methods of assessment, observational studies consistently report higher rates of incident dementia among individuals with higher degrees of frailty^4,26^. Critically, frailty and sensory impairments are interrelated—self-reported and objectively measured hearing and vision loss are prospectively associated with incident frailty in ageing cohorts^27,28^. Previous work demonstrates the persistence of associations of sensory impairments with higher dementia incidence following adjustment for chronological age^29^, but no studies have considered potential confounding by differences in rates of frailty. Clarity could be provided by investigating the temporal co-occurrence of frailty and sensory impairments prior to dementia and testing whether the associations of sensory impairments with dementia vary following consideration of degree of frailty.

The association between hearing loss and dementia appears weak when hearing is measured in midlife^14^; whether it strengthens with accumulating age-related vulnerability has been hypothesised but not directly tested^30^. Though the ACHIEVE trial’s finding that hearing intervention helped only a higher risk subgroup^31^, is consistent with such a mechanism. Should a proportion of the relationship between sensory impairments and incident dementia be confounded by frailty, true dementia population attributable fractions for sensory impairments could be smaller than previously reported, with implications for public health approaches to dementia prevention. We aimed to investigate whether sensory impairments are associated with incident dementia independently of their emergence during broader phenotypic ageing, as identified by a high degree of frailty. By applying continuous-time multi-state Markov models, Cox proportional hazards models with time-varying exposures, and recalculating population-attributable fractions according to the timing of sensory impairment relative to frailty development, we pursued three objectives: (1) determine the temporal relationships between sensory impairments and frailty, including the frequency with which sensory impairments precede, co-occur with, or follow the onset of frailty; (2) quantify the associations of sensory impairments with incident dementia, independent of and jointly with frailty, including whether these associations differ according to their temporal sequence; (3) estimate and compare population attributable fractions for dementia associated with sensory impairments, examining how adjustment for the co-occurrence and temporal sequence with frailty influences the estimated proportion of potentially preventable dementia cases attributable to sensory impairments.

## Results

### Participant characteristics

The analytic cohort comprised 22,756 HRS and 12,921 ELSA participants, the characteristics of whom are shown in Table 1. Participants were slightly older in HRS than in ELSA, by approximately one year, and HRS comprised a greater proportion of female and non-white individuals. The two cohorts diverged most on socioeconomic and lifestyle indicators—compared with HRS participants, ELSA participants had lower levels of education and were more likely to be current smokers and to report alcohol consumption. Frailty index scores at baseline were higher in HRS than in ELSA (mean difference 0.06), with the prevalence of frailty (frailty index ≥ 0.25) in HRS being almost twice the rate in ELSA (25% versus 14%). Self-reported vision impairment was also more common in HRS (22% versus 13%), although self-reported hearing impairment was comparable across cohorts (18%–19%). In total, 187,030 and 76,068 observations and 297,534 and 131,294 person-years of observation time were analysed for HRS and ELSA, respectively (Supplementary Tables 1–4). Across observation periods, incident dementia was detected more frequently in HRS than in ELSA, with an absolute risk three times higher (17% versus 5%) and incidence rates per 100 person-years more than two times higher (1.3 versus 0.5).

### Multi-state Markov analysis

**Fig. 1:**
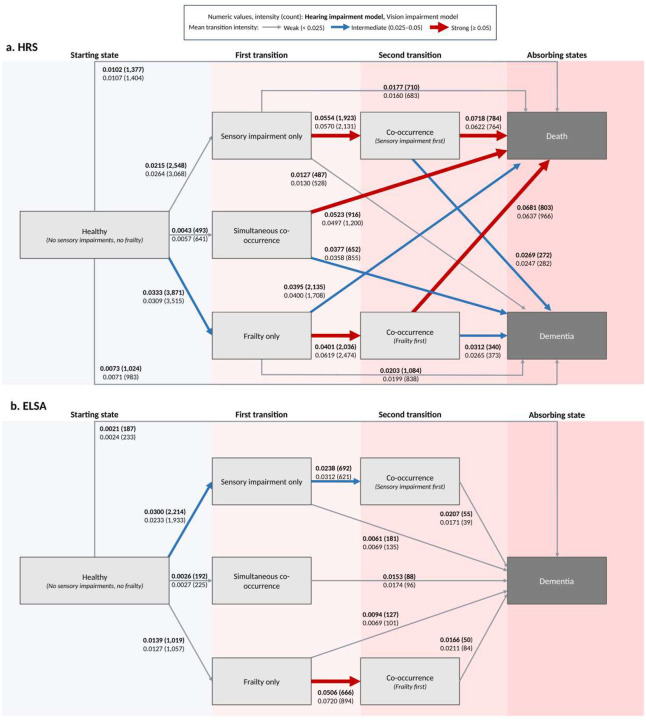
Multi-state model structure and estimated transition intensities for sensory impairment, frailty and dementia in HRS and ELSA. Note: a, HRS, Health and Retirement Study. b, ELSA, English Longitudinal Study of Ageing. Boxes represent states and arrows represent permitted transitions. Participants enter in the starting state (“Healthy”: no sensory impairment and no frailty) and may transition through one of three first-transition states—sensory impairment only, simultaneous co-occurrence, or frailty only—and one of two second-transition states (co-occurrence reached via sensory impairment first, or via frailty first), before reaching the absorbing states. In ELSA (b), dementia is the sole absorbing state because mortality data were not available over the analysis window. Two parallel models were fitted in each cohort, one defining sensory impairment as hearing impairment and one as vision impairment. Arrow labels give the estimated transition intensity (per person-year) with the observed number of transitions in parentheses; bold values correspond to the hearing impairment model and regular values to the vision impairment model. Arrow colour encodes the mean transition intensity across the two models: grey, weak (< 0.025); blue, intermediate (0.025–0.05); red, strong (≥ 0.05). State maintenance (self-transitions from state X to state X) was included in the model for all non-absorbing states but is omitted from the diagram for clarity. Transition intensities were estimated by maximum likelihood under a time-homogeneous continuous-time Markov assumption, with right-censoring at the last observed wave.

To capture how sensory impairments and frailty accumulate over time, we performed continuous-time multi-state Markov models for hearing and vision in each cohort, estimating transition intensities for all permitted transitions between the eight HRS states and the seven ELSA states (Fig. 1). Transition intensities for pathways are summarised below as transition rates per person-year; values and corresponding 95% CIs for all pathways are presented in Supplementary Tables 12–15.

Most participants who developed both a sensory impairment and frailty did so sequentially rather than concurrently. Rates of direct entry from the healthy state into simultaneous co-occurrence of sensory impairment and frailty were low (HRS range 0.004–0.006; ELSA range 0.003–0.003 per person-year). The most frequent first transition from a healthy state was frailty in HRS (range 0.031–0.033, compared with 0.022–0.026 for either sensory impairment), but a sensory impairment in ELSA (0.023–0.030, compared with 0.013–0.014 for frailty). In both cohorts, once either frailty or a sensory impairment had developed, transition rates to co-occurrence were relatively high; rates to co-occurrence from a sensory impairment ranged from 0.055–0.057 in HRS and from 0.024–0.031 in ELSA, while rates to co-occurrence from frailty ranged from 0.040–0.062 in HRS and from 0.051–0.072 in ELSA.

Transition rates to dementia, an absorbing state, were progressively higher in conjunction with an increasing number of conditions present in the starting state. In HRS, rates to dementia increased from healthy (0.007–0.007), sensory impairment only (0.013–0.013), frailty only (0.020–0.020) and co-occurrence (any ordering, 0.025–0.038) states. Similar increases were observed in ELSA: 0.002–0.002 (healthy), 0.006–0.007 (sensory impairment only), 0.007–0.009 (frailty only), 0.015–0.021 (co-occurrence, any ordering). Across both cohorts and both models, the rate of transition to dementia was higher from states in which frailty and a sensory impairment co-occurred than from states with either condition alone, with rate ratios ranging from 1.33 (1.17–1.52) to 3.37 (2.43–4.68) (Supplementary Tables 16 and 17). In HRS, similar gradients were observed for death.

**Fig. 2:**
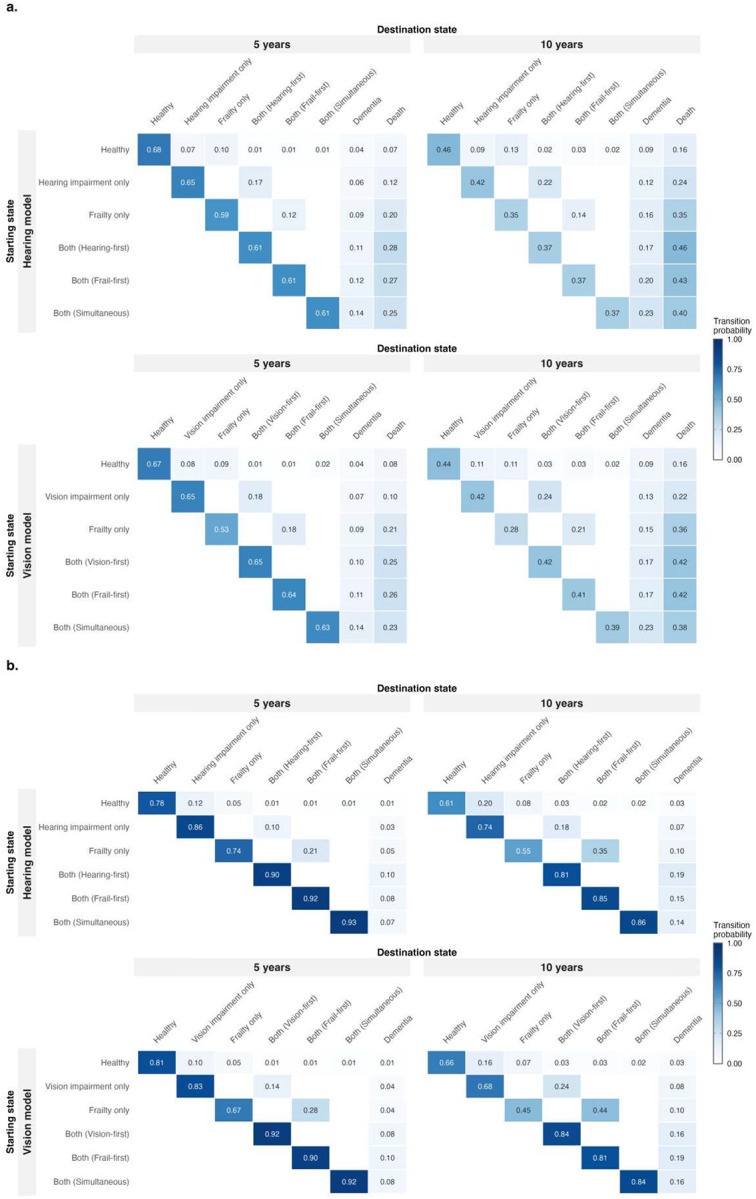
5- and 10-year transition probabilities between health states from the multi-state model in HRS and ELSA Note: a, HRS, Health and Retirement Study. b, ELSA, English Longitudinal Study of Ageing. Each panel shows transition probability matrices at 5 years (left) and 10 years (right) for two parallel multi-state models: one defining sensory impairment as hearing impairment (top row of sub-panels, “Hearing model”) and one defining it as vision impairment (bottom row, “Vision model”). Rows give the starting state at *t* = 0 and columns give the destination state at *t* = 5 or *t* = 10 years; cells therefore give the probability *P* (origin → destination) and each row sums to 1 by construction. States are: Healthy (no sensory impairment, no frailty); single-condition states (hearing or vision impairment only; frailty only); co-occurrence states distinguished by which condition was reached first (sensory-first, frailty-first, or simultaneous within the same wave); and the absorbing states dementia and, in HRS only, death. ELSA does not include death as an absorbing state because mortality data were not available over the analysis window, which is why the corresponding column is absent in b. Cell shading encodes transition probability on the scale shown in the colour bar (0, white → 1, dark blue); numeric values < 0.01 are suppressed for legibility. Probabilities were obtained by exponentiating the maximum-likelihood transition-intensity matrix from the time-homogeneous continuous-time Markov model (*P*(*t*) = exp (*Q t*)) under the assumption of constant intensities between waves.

By 5 years, the probability of remaining in the healthy state was 0.67–0.68 in HRS and 0.78–0.81 in ELSA, reducing to 0.45–0.46 in HRS and 0.61–0.66 in ELSA by 10 years (Fig. 2). At 10 years, the probability of transition directly from the healthy state to dementia was low (0.09 in both HRS models; 0.03 hearing model and 0.08 vision model in ELSA) and increased with the number of conditions present at the starting state, reaching 0.13–0.16 from single-condition states and 0.17–0.25 from co-occurrence states in HRS, and 0.07–0.10 and 0.14–0.19 from the corresponding states in ELSA. Within single-condition states, the 10-year probability of dementia was consistently higher from frailty only than from hearing or vision impairment only in both cohorts. Among co-occurrence states, however, transition probabilities to dementia were similar regardless of which condition was reached first, indicating that the larger co-occurrence effect reflected the presence of both conditions rather than the order in which they accumulated. In HRS, the 10-year probability of death increased from 0.14 (healthy state) to 0.33–0.46 (co-occurrence state).

### Associations of sensory impairments and frailty with incident dementia

To evaluate the associations between sensory impairments, frailty, and dementia risk, we first used Kaplan-Meier cumulative-incidence survival curves and log-rank tests. Curves stratified by baseline impairment status and frailty status diverged early and remained separated across the follow-up period, with consistently higher dementia incidence among participants with sensory impairments or with frailty in both cohorts (Supplementary Figs. 1–6).

**Fig. 3:**
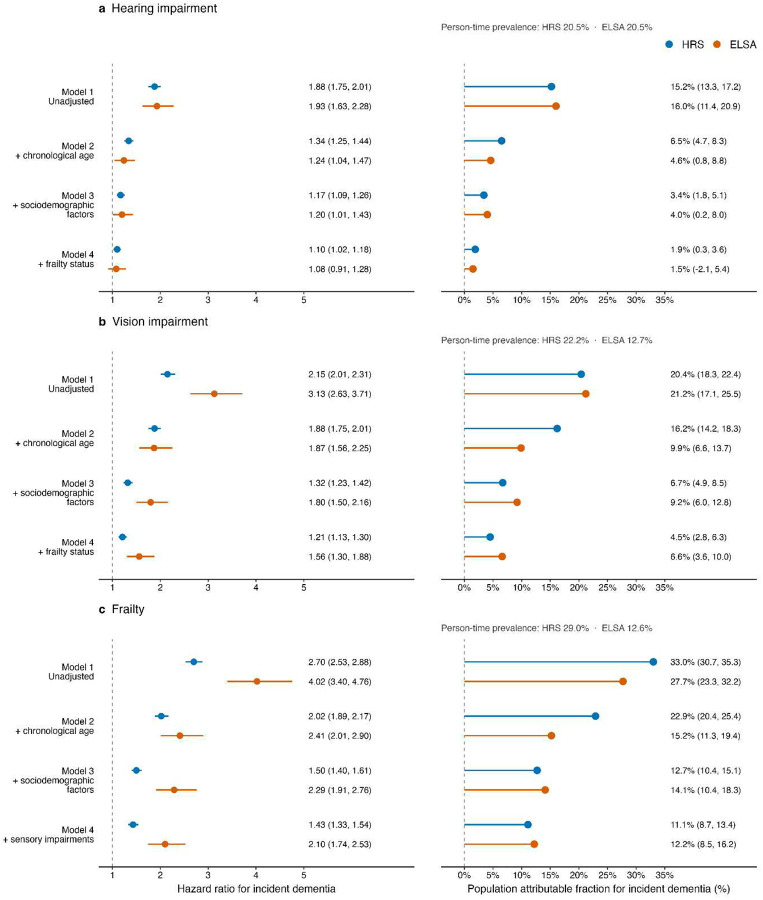
Time-varying associations of hearing impairment, vision impairment and frailty with incident dementia in HRS and ELSA. Note: a, Hearing impairment. b, Vision impairment. c, Frailty. Left, hazard ratios (HR) for incident dementia with 95% confidence intervals (CI); the dashed line denotes HR = 1. Right, population attributable fractions (PAF, %) with 95% CI; the dashed line denotes PAF = 0. Estimates are derived from time-varying Cox proportional hazards models with lagged exposures in HRS, Health and Retirement Study (n = 22,756 participants, 3,877 incident dementia) and ELSA, English Longitudinal Study of Ageing (n = 12,921 participants, 688 incident dementia); PAFs are weighted by person-time exposure prevalence, which is reported above each panel. Model 1, unadjusted; Model 2, adjusted for chronological age; Model 3, additionally adjusted for sex, race/ethnicity, education, marital status, employment, smoking, and alcohol consumption; Model 4, for a and b, Model 3 plus frailty status; for c, a mutually adjusted model including hearing impairment, vision impairment and frailty.

In time-varying Cox proportional hazards models (Fig. 3 and Supplementary Table 18), hazard ratios for incident all-cause dementia for all three exposures attenuated progressively with adjustment for potentially confounding characteristics. In both cohorts, when unadjusted for covariates (Model 1), hazard ratios were highest for frailty (HRS 2.70 [2.53–2.88], ELSA 4.02 [3.40–4.76]), followed by vision impairment (HRS 2.15 [2.01–2.31], ELSA 3.13 [2.63–3.71]) and hearing impairment (HRS 1.88 [1.75–2.01], ELSA 1.93 [1.63–2.28]). Hazard ratios weakened considerably when adjusted for chronological age (Model 2), and—more so in HRS than in ELSA—weakened further when adjusted for additional demographic and lifestyle characteristics (Model 3). Under the fully adjusted Model 4, which additionally included lagged frailty status, hazard ratios associated with hearing impairment reduced to 1.10 (1.02–1.18) in HRS and 1.08 (0.91–1.28) in ELSA—a 41% and 60% reduction in excess risk over Model 3—and hazard ratios associated with vision impairment reduced to 1.21 (1.13–1.30) and 1.56 (1.30–1.88)—a 34% and 30% reduction. Under mutual adjustment for hearing and vision impairment in Model 4, hazard ratios associated with frailty were 1.43 (1.33–1.54) in HRS and 2.10 (1.74–2.53) in ELSA.

Population attributable fractions tracked the same pattern (Fig. 3). Across Models 1–4, the PAF for hearing impairment reduced from 15.2% (13.3–17.2%) to 1.9% (0.3–3.6%) in HRS and from 16.0% (11.4–20.9%) to 1.5% (−2.1 to 5.4%) in ELSA. For vision impairment, PAFs reduced from 20.4% (18.3–22.4%) to 4.5% (2.8–6.3%) in HRS and from 21.2% (17.1–25.5%) to 6.6% (3.6–10.0%) in ELSA. For frailty, it reduced from 33.0% (30.7–35.3%) to 11.1% (8.7–13.4%) in HRS and from 27.7% (23.3–32.2%) to 12.2% (8.5–16.2%) in ELSA.

### Temporal sequencing of frailty and sensory impairment in relation to dementia

**Fig. 4:**
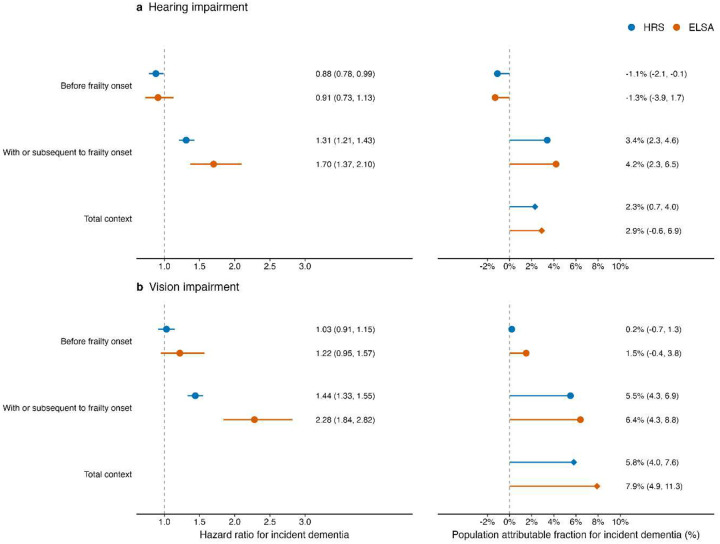
Frailty-stratified associations of hearing and vision impairment with incident dementia in HRS and ELSA. Note: a, Hearing impairment. b, Vision impairment. Estimates compare person-time with and without sensory impairment within two mutually exclusive windows defined by each participant’s current frailty status: before frailty onset (person-time accrued while not yet frail) and with or after frailty onset (person-time accrued from the first frail wave onwards). Window membership is assigned from current, lagged frailty status rather than from whether a participant ever becomes frail, so the before-frailty window includes the not-yet-frail person-time of participants who subsequently become frail; estimates are therefore not restricted to participants who remain non-frail throughout follow-up. Left, hazard ratios (HR) for incident dementia, with 95% confidence intervals (CI), from time-varying Cox proportional hazards models with lagged exposures, fitted separately within each window and adjusted for baseline age, sex, race/ethnicity, education, marital status, employment, smoking and alcohol consumption; the dashed line denotes HR = 1 and the x axis is on a log scale. Right, corresponding population attributable fractions (PAF, %), with 95% CI, weighted by person-time exposure prevalence within each window; the dashed line denotes PAF = 0, and negative values indicate an inverse association. Circles denote window-specific estimates, and “Total context” (diamonds) gives the overall estimate combining the two windows, weighted by the person-time spent in each. HRS (Health and Retirement Study) is shown in blue and ELSA (English Longitudinal Study of Ageing) in orange.

To determine whether associations of sensory impairments with incident dementia differed by temporal sequence with frailty onset, we replaced the binary sensory exposure in Model 5 with a three-level, time-varying variable distinguishing sensory impairment developing before or without frailty onset from sensory impairment developing concurrently with or subsequent to frailty onset (Fig. 4 and Supplementary Table 19).

Time-varying Cox proportional hazards models revealed that associations of sensory impairments with incident all-cause dementia were either considerably weaker or absent if sensory impairments developed without frailty. Relative to no hearing impairment, hazard ratios associated with hearing impairment that developed before or without subsequent frailty were 0.88 (95% CI, 0.78–0.99) in HRS and 0.91 (0.73–1.13) in ELSA, and corresponding hazard ratios associated with hearing impairment that developed concurrently with or subsequent to frailty onset were 1.31 (1.21–1.43) and 1.70 (1.37–2.10). Similarly, relative to no vision impairment, hazard ratios associated with vision impairment that developed before or without subsequent frailty were 1.03 (0.91–1.15) in HRS and 1.22 (0.95–1.57) in ELSA, and corresponding hazard ratios associated with vision impairment that developed concurrently with or subsequent to frailty onset were 1.44 (1.33–1.55) and 2.28 (1.84–2.82).

Disaggregated population attributable fractions paralleled those findings (Fig. 4). For hearing impairment, PAFs were −1.1% (95% CI, −2.1 to −0.1) and −1.3% (−3.9 to 1.7) before or without subsequent frailty onset, 3.4% (2.3 to 4.6) and 4.2% (2.3 to 6.5) concurrently with or subsequent to frailty onset, and 2.3% (0.7 to 4.0) and 2.9% (−0.6 to 6.9) for the total context, in HRS and ELSA, respectively. For vision impairment, the corresponding PAFs were 0.2% (−0.7 to 1.3) and 1.5% (−0.4 to 3.8) before or without subsequent frailty onset, 5.5% (4.2 to 6.8) and 6.4% (4.3 to 8.8) concurrently with or subsequent to frailty onset, and 5.7% (4.0 to 7.5) and 7.9% (4.9 to 11.3) for the total context.

### Age and sex subgroup analyses

Supplementary Fig. 7 presents the associations of hearing impairment, vision impairment and frailty with incident dementia across age and sex subgroups in both cohorts. The direction and statistical significance of the main associations overall (Model 4) and those disaggregated by temporal context with frailty onset (Model 5) were mostly preserved within every stratum. Stronger associations of frailty were observed among participants aged under 65 years in both HRS and ELSA. In HRS, hearing impairment and vision impairment both had stronger associations in participants aged under 65 years, too. In ELSA, vision impairment associations when disaggregated by temporal context with frailty also differed by age: before or without subsequent frailty, vision impairment was only associated with incident dementia in participants aged 65 years or older. In contrast, concurrently with or subsequent to frailty, vision impairment was associated with incident dementia more strongly in participants aged under 65 years. No statistically significant sex interactions were observed.

### Sensitivity analyses

To assess the robustness of our findings, we conducted three pre-specified sensitivity analyses. (1) In HRS, which had available mortality data across the observation window, associations remained mostly consistent after accounting for death as a competing risk through a Fine–Gray subdistribution hazard model (Supplementary Tables 20–21 and Supplementary Figs. 8–9). Here, re-calculated PAFs were slightly higher for hearing loss (2.6% vs. 1.9%), about the same for vision impairment (4.2% vs. 4.5%), and lower for frailty (8.4% vs 11.1%); associations when disaggregated by temporal context with frailty were consistent. (2) We also found similar associations to those of the main analyses in both cohorts when exposures were instead lagged by two waves (~4 years) rather than one wave (~2 years) to mitigate potential reverse causation (Supplementary Tables 22–23 and Supplementary Figs. 10–11). (3) Finally, we recomputed frailty index scores after excluding activities of daily living (ADL) and instrumental-ADL (IADL) items to confirm that the observed associations of frailty with incident dementia were not driven exclusively by functional dependence and found comparable results (Supplementary Tables 24–25 and Supplementary Figs. 12–13). Across all sensitivity analyses, the temporal-context patterns of associations persisted in both cohorts, whereby sensory impairments arising concurrently with or subsequent to frailty onset conferred a higher hazard and a larger PAF than those preceding or without subsequent frailty.

## Discussion

Using population-based data from HRS in the United States and ELSA in the United Kingdom, we show that the association of sensory impairments and incident dementia depends fundamentally on whether the impairment emerges before, alongside, or after the development of frailty. Associations attenuated once age-related vulnerability was considered, and sensory impairments carried no independent association with dementia when they developed in its absence. Instead, they were consistently associated with dementia only when they arose concurrently with, or after, frailty. If sensory impairment itself was a primary driver of dementia, the conferred risk could be hypothesised to be greatest where it runs ahead of systemic decline. However, we show here that the signal emerges only as sensory impairment becomes embedded in advanced multisystem ageing. Considered together, these findings suggest that much of the dementia burden previously attributed to sensory loss may instead reflect the broader ageing-related vulnerability in which sensory deficits frequently accrue.

These findings refine the consistent observational evidence linking sensory impairment to dementia^11,12,13,14^ and the conclusions of the 2024 Lancet Commission on dementia prevention^17^. Hearing and vision loss remain plausible modifiable targets, but their independent contribution to incident dementia is less certain given that their conferral of dementia risk required concurrent or pre-existing frailty. This is consistent with the ACHIEVE randomised controlled trial, in which hearing intervention slowed cognitive decline exclusively among a higher-risk subgroup of older adults^31,32^. From our Markov multi-state analyses, we also found that sensory impairments and frailty often co-occurred, and dementia risk was highest when both developed. Conceptually, this aligns with the position of sensory deficits as components of multisystem deficit accumulation rather than discrete pathological lesions^24,25,33^, and with the broader hypothesis that biological age—better indexed by frailty than by chronological age—captures the ageing context in which other risk factors manifest^22,23^.

The direction of effect modification by frailty in our research is notable because it inverts the pattern previously reported for other dementia risk factors. Associations of Alzheimer pathology and polygenic risk for Alzheimer’s disease with clinical dementia tend to attenuate as frailty rises^26,33^, an observation usually interpreted as evidence that frailty depletes physiological reserve sufficiently to lower the clinical threshold for dementia. In contrast, we found here that sensory impairment carried essentially no excess hazard in the period before frailty had emerged and a substantial, consistent hazard in the period thereafter. One explanation for these findings is that sensory deficits become consequential only once compensatory cognitive and physical reserves have been depleted^34^. This mechanism would also account for the ACHIEVE findings, in which hearing intervention slowed cognitive decline in a subgroup of older adults in whom compensatory reserves were likely already diminished^31,32^.This is compatible with the mechanisms previously proposed for the sensory–dementia link—effortful listening, social withdrawal, accelerated regional brain atrophy and reduced engagement^15,16^—but our data frame them as pathways that require an already-stressed ageing system to translate into clinical decline rather than as independent drivers of dementia risk.

A second substantive finding is the divergence between cohorts in the temporal ordering of frailty and sensory impairment. In HRS, frailty more commonly preceded sensory impairment; in ELSA, sensory impairment more commonly preceded frailty. Several non-mutually exclusive explanations are plausible. Participants from HRS had on average a higher degree of frailty at baseline than their ELSA counterparts (mean frailty index 0.18 versus 0.12), with frailty prevalence almost twice as high (25% versus 14%). For any given trajectory of deficit accumulation, an HRS participant was therefore structurally closer to the 0.25 threshold from the outset; smaller absolute increases in deficit count pushed individuals across the frailty boundary earlier in the observation window. Differences in healthcare access may also be relevant: under the UK NHS, routine sight tests and audiology referrals may detect and frame sensory deficits earlier in the ageing process^35,36^, whereas in HRS the same impairments may be recognised only once they intrude on daily function. Crucially, despite the divergent sequencing, the central finding—that risk concentrates in sensory impairment co-occurring with or following frailty—was preserved in both cohorts, suggesting that the substantive conclusion generalises beyond the particular national context in which it was observed.

Several implications follow for dementia risk reduction strategy. First, PAFs for sensory impairments, calculated without joint consideration of frailty, may overstate the dementia burden that could plausibly be prevented through sensory correction alone, and current estimates of preventable dementia at the population level may need to be revised downward, particularly for hearing impairment. Second, public health interventions targeted exclusively at correction of sensory deficits—for example, large-scale hearing-aid provision—may yield smaller absolute reductions in dementia incidence than headline PAFs imply^31^ unless also accompanied by frailty-focused, multidomain interventions^37,38^, including physical activity and dietary components. Third, the ‘with or subsequent to’ frailty hazard estimates indicate that frailty-informed strategies—combined sensory, functional and behavioural interventions delivered to older adults with established frailty—may identify a population in whom sensory correction could carry larger absolute benefit per intervention^32^. Fourth, these findings reinforce the case for systematic frailty identification in primary care, where older patients’ overall care and multidomain interventions are often coordinated. Embedding frailty measurement into routine primary care would offer a practical route to identifying individuals at substantially elevated dementia risk, particularly when accompanied by their presentation to audiology or optometry services with a new sensory impairment, given how often sensory decline and broader age-related vulnerability coincide^39,40^. These considerations sit alongside recent calls to operationalise dementia prevention as a measurable population-level priority^6,41,42^, and enhance the case for population-level approaches that act on the ageing context in which sensory deficits accumulate.

Our analysis has several strengths. The harmonised application of a single analytic framework to two of the largest and best-characterised population-based global ageing cohorts enabled direct cross-national comparison while preserving cohort-specific structure. Prospective measurement of sensory and frailty status across multiple waves, with validated dementia ascertainment and a deficit-accumulation operationalisation of frailty that captures cumulative multisystem decline^24,25,43^, allowed us to model temporal sequencing directly rather than infer it from baseline correlations. In combination with multi-state Markov modelling, time-varying Cox regression, and disaggregated PAF estimation, we extracted more information about pathway structure than any of these approaches considered in isolation.

It is essential to acknowledge the limitations of our approach. Hearing and vision impairment were ascertained by self-report items rather than by audiometric or visual-acuity testing. Although both the HRS and ELSA have been used in cross-cohort sensory–dementia work^12^, misclassification is inevitable; the expected direction of bias from non-differential misclassification is toward the null, suggesting that the modest pre-frailty associations we observed are unlikely to be substantially larger under objective measurement, and that the concurrent with or subsequent to frailty hazards probably underestimate the true effect. The timing of impairment is also harder to capture than its presence, because sensory loss develops gradually and is under-reported among older adults. Recorded onset may therefore lag true onset more frequently at older ages, misassigning some pre-frailty impairments to the concurrent or subsequent category^44^. Frailty was modelled as a binary status defined at the conventional 0.25 threshold^22,45^. This treatment is appropriate to a question framed around temporal sequencing but cannot represent sub-threshold acceleration in deficit accumulation. Continuous frailty trajectories may identify further heterogeneity in dementia risk among individuals who never crossed the binary threshold, and future work using slope-based or trajectory-based exposures is warranted to determine whether the temporal dependency observed here extends to pre-clinical phases of frailty. Dementia ascertainment differed between cohorts (Langa–Weir cognitive testing in HRS^46^; physician diagnosis combined with the IQCODE in ELSA^47^). The Langa–Weir algorithm is based on the telephone-administered TICS—immediate and delayed word recall, serial subtraction and backward counting—tasks that depend on hearing the examiner, so participants with hearing impairment may score lower for reasons unrelated to cognition. This differential misclassification would inflate the hearing–dementia association in HRS specifically. Despite this, the consistency of associations across cohorts argues against ascertainment differences as a driver of the central finding. Absolute dementia incidence was approximately three times higher in HRS than in ELSA (17% versus 5%), a gap likely to reflect ascertainment differences alongside differences in baseline frailty and follow-up duration. Finally, the observational design cannot rule out residual confounding from unmeasured ageing-related processes and our findings should be interpreted as quantifying associations under a specified analytic framework, not as estimates of intervention effects.

In conclusion, our analysis suggests that the association between sensory impairment and incident dementia is largely conditional on the prior or concurrent emergence of frailty. The implication for dementia prevention is not that sensory impairments should be deprioritised—they remain among the most accessible and most easily corrected age-related conditions—but that estimates of their preventable contribution to dementia, and the populations in whom intervention is most likely to be effective, should be revisited in light of the broader ageing context within which sensory impairments occur.

## Methods

### Study design and data sources

We performed a harmonised longitudinal analysis of observational data from two nationally representative cohorts of community-dwelling older adults: the U.S. Health and Retirement Study (HRS) and the English Longitudinal Study of Ageing (ELSA). Ethical approval for the HRS was granted by the Health Sciences and Behavioural Sciences Institutional Review Board of the University of Michigan, and for ELSA by the National Health Service (NHS) Research Ethics Committees under the National Research Ethics Service. Written informed consent was obtained from all participants at each wave of data collection. This study constitutes a secondary analysis of de-identified data; patients and the public were not involved in its design or conduct. Reporting adheres to the Strengthening the Reporting of Observational Studies in Epidemiology (STROBE) Statement.

The HRS is an ongoing biennial panel study of adults in the United States aged 50 years or older and their spouses, established in 1992, with refresher cohorts periodically introduced to maintain population representativeness. At each wave, participants completed core interviews covering demographics, physical and cognitive function, chronic conditions, and health behaviours. For the present analysis, we used harmonised variables from the RAND HRS Products, linked to cognitive status derived using the Langa–Weir classification. We selected the 2004 wave as the rolling baseline start wave for HRS given that it marked the availability of data relating to frailty indices and follow-up ended at the 2020 wave. HRS data can be requested at https://hrs.isr.umich.edu/.

ELSA is an ongoing biennial panel study of community-dwelling adults aged 50 years or older living in England, initiated in 2002 using the Health Survey for England as the sampling frame, with refresher cohorts added at follow-up waves. Information was obtained through computer-assisted interviews and self-completion questionnaires at each wave, supplemented by nurse visits in alternating waves that yielded objective measures including anthropometry, blood pressure and blood samples. We used data from ELSA waves 1–9 (2002–2018/19), drawing covariate, sensory, and frailty items from the core interview and derived-variables files; incident dementia was ascertained from self- or proxy-reported physician diagnosis, supplemented by the Informant Questionnaire on Cognitive Decline in the Elderly (IQCODE). ELSA data can be requested at https://ukdataservice.ac.uk/.

To fully construct comparable analytic samples across the two cohorts, we adopted a rolling-baseline design in which baseline was defined as the earliest wave within 2004–2010 (HRS) or 2002–2008 (ELSA) at which a participant first met all eligibility criteria. Participants were eligible if, at the baseline wave, they were aged 50 years or older, were free from dementia, and had non-missing data on hearing impairment, vision impairment, frailty, and analytical covariates (age, sex, education level, marital status, and ethnicity). They were also required to have at least one follow-up wave with non-missing measurements on frailty, hearing impairment, vision impairment, and dementia status. Applying these criteria yielded an analytic cohort of 22,756 HRS participants and 12,921 ELSA participants. Full exclusion flows, including dementia cases lost at each step, are reported in Supplementary Tables 1–4.

### Assessment of sensory impairment

The status of sensory impairments was ascertained at each wave from harmonised self-report hearing and vision items in both HRS and ELSA, consistent with prior cross-cohort analyses of sensory function in older adults^12^. Both items were administered with the instruction for participants to respond in relation to their level of function as gauged while using hearing aids or corrective lenses as usual. Exposures therefore reflected residual rather than uncorrected impairment, isolating functional deficits that plausibly remain on pathways to dementia. Hearing impairment and vision impairment were analysed as two separate binary exposures (no impairment, impairment). Sensory status was reassessed at every biennial wave (HRS, 2004–2020; ELSA, waves 1–9, 2002–2018/19), and the onset of each impairment was defined as the first wave at which the participant met the impairment criterion, and as time-varying in longitudinal analyses to capture transitions between unimpaired and impaired states across follow-up.

Hearing was assessed with a single question in which participants rated their hearing as “excellent”, “very good”, “good”, “fair”, or “poor”. Following the operationalisation widely adopted in HRS and ELSA, participants responding “fair”, or “poor” were classified as having hearing impairment, and those responding “excellent”, “very good”, or “good” as having no hearing impairment. Vision was assessed by an analogous question on self-rated eyesight, offering the same five response options together with an additional category recorded as “legally blind” in HRS and “registered with local council as sight impaired” in ELSA. Participants responding “fair”, “poor”, or within the additional category were classified as having vision impairment, and those responding “excellent”, “very good”, or “good” as having no vision impairment. Non-substantive responses (“don’t know”, “refused”, “not applicable”) were set to missing in both cohorts. A full description of sensory impairments’ items is provided in Supplementary Table 5.

### Frailty measurement

Frailty was assessed using a deficit-accumulation frailty index calculated retrospectively in each cohort in accordance with standard procedure^43^. The frailty index is a measure of overall health state that integrates information from multiple physiological systems and closely reflects an individual’s risk of adverse health events and mortality independently of chronological age^24,25^. The deficits comprising a frailty index are routinely collected clinical and self-report items—symptoms, signs, disabilities and diseases—that meet established criteria for inclusion. Because frailty index scores capture the cumulative burden of age-related deficits across systems, they are increasingly used as a quantitative marker of biological age and have been shown to track biological ageing more closely than chronological age alone^48,49^.

Both the HRS and ELSA frailty indices have been derived and validated previously^50,51,52^. In HRS, the frailty index comprised 37 health and functional deficits, spanning basic and instrumental activities of daily living, mobility, diagnosed chronic conditions, depressive and related affective symptoms from the 8-item Center for Epidemiologic Studies Depression (CES-D) scale, self-rated health, underweight status, pain, and urinary incontinence. In ELSA, the frailty index comprised 49 deficits, covering mobility limitations, activities of daily living, diagnosed chronic conditions, the 7-item ELSA CES-D scale, self-rated general health, falls, hip fracture, joint replacement, and pain. In both cohorts, deficits were coded as present (1) or absent (0), except for self-rated health, which was graded on a five-level scale (0, 0.25, 0.50, 0.75, 1). As our primary outcome was incident dementia, each frailty index was modified to exclude deficits closely related to hearing, vision, and cognition, consistent with previous work^4^.

For each participant at each wave, frailty index score was computed as the ratio of accumulated deficits to non-missing items, yielding a continuous score from 0 to 1, where higher values indicate accumulation of more age-related health deficits and a greater degree of frailty, and was calculated only for participants with valid information on at least 80% of the constituent deficits (≥ 30 items for HRS; ≥ 40 items for ELSA). Participants were categorised as frail (frailty index score ≥ 0.25) or non-frail (frailty index score < 0.25) using the threshold established in prior cohort studies of older adults^45,53^. Frailty status was updated at each biennial wave as well, with frailty onset defined as the first wave at which the frailty index reached ≥ 0.25, providing the corresponding time points for the frailty exposure. The full list of frailty index deficits in each cohort is provided in Supplementary Table 6.

### Incident dementia

The primary outcome was incident all-cause dementia, ascertained at each wave using validated, cohort-specific algorithms previously adopted in HRS and ELSA^46,47^. In HRS, dementia was ascertained using the Langa–Weir classification, which integrates a 27-point composite score from the Telephone Interview for Cognitive Status (TICS) of immediate and delayed word recall, serial seven subtraction, and backward counting. TICS scores of 0–6 indicate the presence of dementia. In ELSA, dementia was ascertained from two complementary sources—a self- or proxy-reported physician diagnosis of Alzheimer’s disease or dementia, and the 16-item Informant Questionnaire on Cognitive Decline in the Elderly (IQCODE). Mean IQCODE scores ≥ 3.4 (computed from at least 10 valid items) indicate a decline in everyday function over the preceding two years consistent with dementia^4^. Participants meeting either ELSA criterion were classified as cases of dementia. In both cohorts, dementia was treated as an absorbing state and carried forward to all subsequent waves.

### Covariates and mortality

Covariates were selected a priori on the basis of their established associations with sensory function, frailty, and/or dementia in older adults, and were harmonised across both cohorts^54,55^. They were handled in two strata according to whether they were treated as fixed at baseline or updated at each follow-up wave. Time-invariant covariates were set at each participant’s baseline wave and comprised age (in years, modelled continuously), sex (male or female), self-reported race or ethnicity (dichotomised as White or non-White), educational attainment (categorised as lower, intermediate, or higher), and marital status (married or partnered versus separated, divorced, widowed, or never married). Time-varying covariates were updated at each biennial wave and comprised employment status (currently working or retired versus not in the labour force), smoking status (current smoker versus not), and alcohol consumption (any drinking days versus none). Cohort-specific source variables and response-option mappings were harmonised to common categories across HRS and ELSA; a full description of the source items and coding rules is provided in Supplementary Table 7.

Mortality data were incorporated into analyses where applicable. For HRS, mortality was ascertained through linkage to the National Death Index and supplemented by the HRS exit interview, providing complete mortality follow-up across the observation window. For ELSA, linked mortality data were available only through Wave 6 (2012); although more recent records exist, data-sharing agreements restrict their access to the ELSA core team. Mortality was therefore not modelled as a separate state in ELSA analyses, and analyses requiring complete mortality follow-up (specifically, the eight-state multi-state model and the competing-risks sensitivity analysis) were conducted in HRS only.

### Statistical analyses

All analyses were performed in R version 4.4.0 with HRS and ELSA analysed separately. Two-sided P values below 0.05 were considered statistically significant.

### Descriptive analyses and missingness

Rolling-baseline characteristics of the analytic cohorts were summarised as means with standard deviations for continuous variables and as counts and percentages for categorical variables (Table 1). Missingness across covariates was low (approximately 1–10% per variable, depending on the wave) and showed no discernible pattern suggestive of systematic loss to follow-up. Missing values in the time-varying and time-invariant covariates were therefore imputed using multiple imputation by chained equations (MICE) under a fully conditional specification, implemented in the *“mice”* R package^56^. Separate imputation models were fit at each wave of each cohort, using predictive mean matching for continuous variables, proportional-odds ordinal regression for ordered categorical variables (educational level), and logistic regression for binary variables. Twenty imputed datasets were generated with ten iterations each; convergence statistics was assessed, and the imputed data were compared against original data using summary statistics. The primary study variables—frailty index scores, sensory-impairment status, and dementia status—were not imputed.

### Multi-state Markov modelling (objective 1)

To characterise the temporal sequence through which sensory impairments, frailty, and dementia arise (objective 1), we fitted a continuous-time, time-homogeneous Markov multi-state model separately for hearing impairment and vision impairment using the *“msm”* R package^57^. At each wave, participants were assigned to one of a set of mutually exclusive states, defined by the joint occurrence and temporal ordering of sensory impairment and frailty: (1) no sensory impairment and no frailty (healthy); (2) sensory impairment only; (3) frailty only; (4) sensory impairment followed by frailty; (5) frailty followed by sensory impairment; (6) simultaneous onset of sensory impairment and frailty in the same wave; and (7) dementia. The order of onset distinguishing states 4 and 5 was established from the earliest wave at which each condition was recorded. Once a participant entered a state, time was measured continuously from the baseline wave, and the participant remained in that state until the next wave at which reclassification (or state maintenance) was triggered. For HRS, death was incorporated as an additional absorbing state (state 8), yielding an eight-state model in which both dementia and death were specified as absorbing. For ELSA, mortality data were not available across the full follow-up window, and a seven-state model was fitted with dementia as the sole absorbing state. This parallel structure is a recognised approach in multi-state analyses of ageing cohorts when complete mortality follow-up cannot be assumed^58,59^. Full state definitions and permitted transitions for each cohort are detailed in Supplementary Tables 8–11.

Transitions were encoded in the transition-intensity matrix $Q=\left\{q_{ij}\right\}$, where $q_{ij}\geq 0$ denotes the instantaneous rate of transition from state *i* to state $j\left(i\neq j\right)$. Transitions not represented in the conceptual model—for example, reversals from a “both” state back to a single-condition state, or direct jumps from state 1 to states 4 or 5 bypassing the single-condition intermediate—were fixed at zero. Because repeated participant assessments occurred at discrete biennial waves while state changes arise in continuous time, a time-homogeneous model was preferred instead of a discrete one, and all transitions were treated as interval censored. The likelihood was maximised using the forward algorithm, with initial values generated internally from the observed state-transition frequencies. From the fitted models, we derived (with 95% confidence intervals) ratios of competing transition intensities—for example, the relative rate of progression to dementia from the “sensory-first” intermediate state versus the “frailty-first” intermediate state, and the transition-probability matrix evaluated at *t* = 5 and 10 years.

### Evaluation of associations between sensory impairments, frailty, and incident dementia (objective 2)

The unadjusted association between baseline sensory status, frailty index scores and incident dementia were first visualised using Kaplan–Meier cumulative-incidence curves, stratified by hearing- and vision-impairment status, and frailty status at baseline and compared using the log-rank test (Supplementary Figs. 1–6). To analyse the associations of sensory impairments and frailty with incident dementia, we fitted Cox proportional hazards models in a counting-process formulation with time-varying exposures, in which follow-up time for each participant was split into successive biennial intervals bounded by consecutive interview waves. All time-varying independent variables—hearing impairment, vision impairment, frailty status, and the lifestyle covariates (employment, smoking status, and alcohol consumption)—were entered as one-wave lagged values measured at the start of each interval, meaning that exposure status at the beginning of the interval predicted dementia onset during it. This design avoids conditioning on post-exposure information and attenuates the risk of reverse causation arising from pre-clinical cognitive decline influencing the exposure assessment^60^.

We fitted a pre-specified series of nested Cox proportional hazards models for hearing impairment, vision impairment and frailty status. To ensure that hazard ratios were directly comparable across specifications, all models were fitted on a common analytic sample defined by complete data on every covariate entering Model 4. First, an unadjusted model estimated the crude association between time-varying sensory impairment, frailty status and incident dementia (Model 1). Next, we iteratively added covariates to model 1 to assess how their inclusion altered the strength of relationships between sensory impairments, frailty and incident dementia: Model 2 further adjusted for chronological age; Model 3 additionally included sex, self-reported race or ethnicity, educational level, marital status, and lagged employment, lagged smoking status and alcohol consumption; and Model 4, the primary fully adjusted specification, additionally adjusted for lagged frailty status. To estimate a hazard ratio for frailty that is comparable in scale to the sensory hazard ratios while accounting for their co-occurrence of sensory impairments—the frailty-row Model 4 was instead a mutually adjusted Cox model that simultaneously included lagged hearing impairment, lagged vision impairment, and lagged frailty status alongside all Model 3 covariates. Model 5 was the primary model for pathway inference, in which we replaced the binary sensory-impairment exposure with a three-level time-varying variable capturing its temporal relationship with frailty: level 1, no sensory impairment (reference); level 2, sensory impairment occurring in the absence of frailty up to the start of the interval; and level 3, sensory impairment with concurrent or prior frailty at the start of the interval. The three-level variable was constructed prospectively using only information available up to the start of each interval, thereby precluding any leakage of future exposure or outcome status into the classification and was lagged in the same way as the other time-varying terms. Model 5 retained the Model 3 covariate set, and the within-exposed contrast—the hazard ratio for level 3 relative to level 2—was recovered from a linear hypothesis on the log-hazard scale using the model coefficient covariance matrix, providing a direct test of whether the dementia hazard associated with sensory impairment depends on the prior emergence of frailty.

Ties in event times were handled using the Efron approximation, and robust standard errors accounting for within-participant correlation across intervals were obtained by clustering on the participant identifier. The proportional-hazards assumption was tested for each covariate using scaled Schoenfeld residuals, and corresponding plots were inspected visually for time-dependent departures from proportionality.

### Population attributable fractions (objective 3)

The proportion of incident dementia attributable to sensory impairment was estimated as the population attributable fraction (PAF), using the Levin formulation applied to the adjusted hazard ratios from the Cox models^17,18,61^. For binary exposures, $PAF=\frac{p\left(HR-1\right)}{\left[1+p\left(HR-1\right)\right]}$, where *p* denotes the prevalence of the exposure and HR the adjusted hazard ratio. Because exposure status varied across follow-up, *p* was calculated as the person-time-weighted prevalence—the proportion of total person-time contributed by intervals classified as exposed—so that the prevalence basis and the HR were derived from the same exposure accounting. Binary PAFs were computed sequentially across Models 1–4 to characterise how the estimated population impact of sensory impairment changed with progressive adjustment. This sequence allowed us to quantify the extent to which the apparent population impact of sensory impairment was attributable to its co-occurrence with frailty. For the three-level “context” exposure fit in Model 5, the total PAF was computed as ${PAF}_{\text{total}}=\frac{\sum_{k}p_{k}\left({HR}_{k}-1\right)}{1+\sum_{k}p_{k}\left({HR}_{k}-1\right)}$ and disaggregated into pathway-specific ${PAF}_{k}=\frac{p_{k}\left({HR}_{k}-1\right)}{1+\sum_{j}p_{j}\left({HR}_{j}-1\right)}$, where *k* indexes the exposed strata (sensory impairment occurring in the absence of frailty; sensory impairment with concurrent or prior frailty) and *p*_*k*_ denotes the person-time-weighted prevalence of each stratum. 95% confidence intervals for all PAF estimates were calculated by Monte Carlo simulation: for binary exposures (models 1–4), 10,000 draws were taken from the univariate normal sampling distribution of the log-hazard ratio; for the multi-level exposure (model 5), 10,000 draws of the full log-hazard-ratio vector were taken from its estimated multivariate-normal sampling distribution, preserving the correlation structure among the stratum-specific coefficients. Each draw was transformed to the hazard-ratio scale, and the PAF formulas above were evaluated at each draw to yield empirical 2.5th and 97.5th percentile intervals.

### Subgroup analyses and sensitivity analyses

The robustness of the primary findings was examined through a series of pre-specified sensitivity analyses, conducted in both cohorts where feasible and restricted to HRS where the absence of linked mortality data after Wave 6 or the small number of incident dementia cases in ELSA precluded estimation. First, subgroup analyses were performed in both cohorts to assess effect modification across clinically meaningful strata, defined by baseline age (< 65 versus ≥ 65 years) and sex (male versus female). Second, to address the possibility that ADL and IADL deficits within the frailty index act as mediators rather than confounders of the sensory impairment dementia relationship, the Cox risk analyses (Models 1–5) were re-estimated using a modified frailty index from which all (I)ADL items had been removed. The frailty threshold of ≥ 0.25 was reapplied to the modified index, and the temporal classification underpinning Model 5 was reconstructed accordingly. This sensitivity analysis was not extended to the multi-state Markov model. Third, to account for the possibility that death before dementia diagnosis competes with the event of interest, we re-estimated the HRS Cox models using the Fine–Gray sub-distribution hazard framework with death from any cause specified as the competing event. Finally, to assess the influence of prevalent but undiagnosed dementia on the exposure–outcome association, the primary models were re-estimated in both cohorts after excluding dementia events occurring within the first two waves (4 years) of follow-up.

## Supplementary Material

Supplementary Files

This is a list of supplementary files associated with this preprint. Click to download.

- ZhouNatAgingSupplementaryinformation.docx

## Figures and Tables

**Table 1.** Characteristics of analysed participants

Characteristics	HRS	ELSA
**Total, n**	22,756	12,921
**Age at baseline, years, mean (s.d.)**	63.5 (10.3)	62.5 (9.7)
Range	50–100	50–99
**Sex, n (%)**		
Male	9,631 (42)	5,832 (45)
Female	13,125 (58)	7,089 (55)
**Race/ethnicity, n (%)**		
White	15,439 (68)	12,500 (97)
Non-white	7,308 (32)	395 (3)
**Frailty index score, mean (s.d.)**	0.18 (0.15)	0.12 (0.13)
Non frail (score < 0.25), n (%)	16,994 (75)	11,074 (86)
Frail (score ≥ 0.25), n (%)	5,762 (25)	1,847 (14)
**Education, n (%)**		
Lower	4,456 (20)	4,499 (35)
Intermediate	7,886 (34)	3,855 (30)
Higher	10,414 (46)	4,567 (35)
**Marital status, n (%)**		
Others (separated, divorced, widowed, or never married)	7,375 (32)	4,008 (31)
Married	15,381 (68)	8,913 (69)
**Employment, n (%)**		
Not in labour force	12,052 (53)	7,564 (59)
Currently working or retired	10,704 (47)	5,357 (41)
**Smoking status, n (%)**		
Non-smoker	18,856 (83)	9,429 (73)
Current smoker	3,900 (17)	3,492 (27)
**Alcohol consumption, n (%)**		
Non-drinker	14,362 (63)	4,565 (35)
Any drinking days	8,394 (37)	8,356 (65)
**Hearing impairment, n (%)**		
No	18,546 (82)	10,452 (81)
Yes	4,210 (18)	2,469 (19)
**Vision impairment, n (%)**		
No	17,803 (78)	11,217 (87)
Yes	4,953 (22)	1,704 (13)
**Follow-up**		
**Time, years, mean (s.d.)**	13.1 (4.0)	10.2 (4.9)
**No dementia, n (%)**	18,879 (83)	12,233 (95)
**Incident dementia, n (%)**	3,877 (17)	688 (5)
**Dementia incidence rate per 100 person-years (total person-years follow-up)**	1.3 (297,534)	0.5 (131,294)

Note: s.d. standard deviation; HRS, Health and Retirement Study; ELSA, English Longitudinal Study of Ageing; Values for race/ethnicity were missing for 26 participants in ELSA and 9 participants in HRS.

## Data Availability

The HRS and ELSA data that support the findings are publicly available to registered or approved researchers from the respective cohort custodians but are not redistributable by the authors, in accordance with the data providers’ conditions of use and participant-confidentiality requirements. Health and Retirement Study (HRS) data are distributed by the University of Michigan and were downloaded, following free registration, from the HRS data portal (https://hrsdata.isr.umich.edu/data-products); the products used comprised the HRS Cross-Wave Tracker File (2022 Tracker Final Version 1.0; https://hrs.isr.umich.edu/news/data-announcements/tracker-file-2022-tracker-final-version-10), the RAND HRS products, and the Langa–Weir cognitive classification used to ascertain dementia. Mortality status was derived from the HRS Tracker File and exit interviews. English Longitudinal Study of Ageing (ELSA) data (waves 1–9, 2002–2018/19) are distributed by the UK Data Service (Study Number 5050; https://ukdataservice.ac.uk/) and can be requested following registration and acceptance of the End User Licence.

## Code availability

All analyses were performed in R (version 4.4.0). Data were imported and managed using the haven and labelled packages together with the *tidyverse* suite (including *dplyr*, *tidyr*, *stringr* and *lubridate*). Multiple imputation by chained equations was implemented with *mice*; continuous-time multi-state Markov models were fitted with *msm*; and time-varying Cox proportional hazards models, Kaplan–Meier estimators with log-rank tests, and Fine–Gray competing-risks models were fitted with *survival*, with survival curves and figures produced using *survminer* and scales. Population attributable fractions, including the disaggregated temporal-context estimates, were computed using custom R scripts based on the Levin formulation. The custom analysis code supporting the findings of this study is available https://github.com/SyronZhou/SZ_et_al_Nature_Aging_2026.

## References

[1] GBD 2019 Dementia Forecasting Collaborators. Estimation of the global prevalence of dementia in 2019 and forecasted prevalence in 2050: an analysis for the Global Burden of Disease Study 2019. Lancet Public Health 7, e105–e125 (2022). 34998485 10.1016/S2468-2667(21)00249-8 PMC8810394

[2] Schneider J. A. & Agrawal S. Vascular pathology and pathogenesis of cognitive impairment and dementia in older adults. Cereb. Circ. Cogn. Behav. 3, 100148 (2022). 36324408 10.1016/j.cccb.2022.100148 PMC9616381

[3] Nichols E., Merrick R., Hay S. I. The prevalence, correlation, and co-occurrence of neuropathology in old age: harmonisation of 12 measures across six community-based autopsy studies of dementia. Lancet Healthy Longev. 4, e115–e125 (2023). 36870337 10.1016/S2666-7568(23)00019-3 PMC9977689

[4] Ward D. D. Frailty trajectories preceding dementia in the US and UK. JAMA Neurol. 82, 61–71 (2025). 39527039 10.1001/jamaneurol.2024.3774 PMC11555573

[5] Nonino F. Amyloid-beta-targeting monoclonal antibodies for people with mild cognitive impairment or mild dementia due to Alzheimer’s disease. Cochrane Database Syst. Rev. 4, CD016297 (2026). 41985900 10.1002/14651858.CD016297 PMC13082890

[6] Walsh S. The principles of Population-Level Approaches to Dementia Risk Reduction (PLADRR). PLOS Med. 23, e1005059 (2026). 42048404 10.1371/journal.pmed.1005059 PMC13123963

[7] Zhou S. Association of cumulative average sensory impairments with cognitive function and depressive symptoms: two prospective cohort studies. J. Affect. Disord. 369, 16–24 (2025). 39321973 10.1016/j.jad.2024.09.140

[8] Deal J. A. Hearing impairment and incident dementia and cognitive decline in older adults: the Health ABC Study. J. Gerontol. A Biol. Sci. Med. Sci. 72, 703–709 (2017). 27071780 10.1093/gerona/glw069 PMC5964742

[9] Myrstad C. Hearing impairment and risk of dementia in The HUNT Study (HUNT4 70+): a Norwegian cohort study. eClinicalMedicine 66, 102319 (2023). 38192588 10.1016/j.eclinm.2023.102319 PMC10772264

[10] Osler M., Christensen G. T., Mortensen E. L. Hearing loss, cognitive ability, and dementia in men age 19–78 years. Eur. J. Epidemiol. 34, 125–130 (2019). 30306425 10.1007/s10654-018-0452-2

[11] Cantuaria M. L., Pedersen E. R., Waldorff F. B. Hearing loss, hearing aid use, and risk of dementia in older adults. JAMA Otolaryngol. Head Neck Surg. 150, 157–164 (2024). 38175662 10.1001/jamaoto.2023.3509 PMC10767640

[12] Maharani A., Dawes P., Nazroo J., Tampubolon G. & Pendleton N. Visual and hearing impairments are associated with cognitive decline in older people. Age Ageing 47, 575–581 (2018). 29697748 10.1093/ageing/afy061

[13] Zhang X. Association between sensory impairment and cognitive frailty among older people: evidence from four nationwide cohort studies. J. Nutr. Health Aging 29, 100590 (2025). 40441121 10.1016/j.jnha.2025.100590 PMC12402417

[14] Machado-Fragua M. D. Association of midlife hearing impairment and hearing aid use with incident dementia: analysis of two UK-based longitudinal cohort studies. Nat. Aging 5, 1732–1738 (2025). 40595016 10.1038/s43587-025-00914-1 PMC12443597

[15] Griffiths T. D. How can hearing loss cause dementia? Neuron 108, 401–412 (2020). 32871106 10.1016/j.neuron.2020.08.003 PMC7664986

[16] Vodyanyk M. M., Jaeggi S. M. & Seitz A. R. Multidimensional relationships between sensory perception and cognitive aging. Front. Aging Neurosci. 16, 1484494 (2024). 39759398 10.3389/fnagi.2024.1484494 PMC11695427

[17] Livingston G. Dementia prevention, intervention, and care: 2024 report of the Lancet standing Commission. Lancet 404, 572–628 (2024). 39096926 10.1016/S0140-6736(24)01296-0

[18] Stephan B. C. M. Population attributable fractions of modifiable risk factors for dementia: a systematic review and meta-analysis. Lancet Healthy Longev. 5, e406–e421 (2024). 38824956 10.1016/S2666-7568(24)00061-8 PMC11139659

[19] Tan B. K. J., Man R. E. K., Gan A. T. L., Fenwick E. K., Varadaraj V., Swenor B. K. Is sensory loss an understudied risk factor for frailty? A systematic review and meta-analysis. J. Gerontol. A Biol. Sci. Med. Sci. 75, 2461–2470 (2020). 32735331 10.1093/gerona/glaa171

[20] López-Otín C., Blasco M. A., Partridge L., Serrano M. & Kroemer G. Hallmarks of aging: an expanding universe. Cell 186, 243–278 (2023). 36599349 10.1016/j.cell.2022.11.001

[21] Goh J., Wong E., Soh J., Maier A. B. & Kennedy B. K. Targeting the molecular & cellular pillars of human aging with exercise. FEBS J. 290, 649–668 (2023). 34968001 10.1111/febs.16337

[22] Kim D. H. & Rockwood K. Frailty in older adults. N. Engl. J. Med. 391, 538–548 (2024). 39115063 10.1056/NEJMra2301292 PMC11634188

[23] Diebel L. W. M. & Rockwood K. Determination of biological age: geriatric assessment vs biological biomarkers. Curr. Oncol. Rep. 23, 104 (2021). 34269912 10.1007/s11912-021-01097-9 PMC8284182

[24] Mitnitski A. B., Mogilner A. J. & Rockwood K. Accumulation of deficits as a proxy measure of aging. ScientificWorldJournal 1, 323–336 (2001). 12806071 10.1100/tsw.2001.58 PMC6084020

[25] Rockwood K. A frailty index based on deficit accumulation quantifies mortality risk in humans and in mice. Sci. Rep. 7, 43068 (2017). 28220898 10.1038/srep43068 PMC5318852

[26] Gao P.-Y. Physical frailty, genetic predisposition, and incident dementia: a large prospective cohort study. Transl. Psychiatry 14, 212 (2024). 38802408 10.1038/s41398-024-02927-7 PMC11130190

[27] Kawaguchi K. Self-reported hearing and vision impairment and incident frailty in Japanese older people: a 3-year longitudinal analysis of the Japan Gerontological Evaluation Study. Arch. Gerontol. Geriatr. 104, 104834 (2023). 36257161 10.1016/j.archger.2022.104834

[28] Hou T., Liu M. & Zhang J. Bidirectional association between visual impairment and frailty among community-dwelling older adults: a longitudinal study. BMC Geriatr. 22, 672 (2022). 35971062 10.1186/s12877-022-03365-0 PMC9377125

[29] Yu R.-C., Proctor D., Soni J., Pikett L. Adult-onset hearing loss and incident cognitive impairment and dementia — a systematic review and meta-analysis of cohort studies. Ageing Res. Rev. 98, 102346 (2024). 38788800 10.1016/j.arr.2024.102346

[30] Reed N. S., Huang A. R. & Coresh J. Midlife hearing loss and dementia risk. Nat. Aging 5, 1654–1656 (2025). 40903651 10.1038/s43587-025-00955-6

[31] Lin F. R. Hearing intervention versus health education control to reduce cognitive decline in older adults with hearing loss in the USA (ACHIEVE): a multicentre, randomised controlled trial. Lancet 402, 786–797 (2023). 37478886 10.1016/S0140-6736(23)01406-X PMC10529382

[32] Pike J. R. Cognitive benefits of hearing intervention vary by risk of cognitive decline: a secondary analysis of the ACHIEVE trial. Alzheimers Dement. 21, e70156 (2025). 40369891 10.1002/alz.70156 PMC12078761

[33] Wallace L. M. K. Investigation of frailty as a moderator of the relationship between neuropathology and dementia in Alzheimer’s disease: a cross-sectional analysis of data from the Rush Memory and Aging Project. Lancet Neurol. 18, 177–184 (2019). 30663607 10.1016/S1474-4422(18)30371-5 PMC11062500

[34] Stern Y. Cognitive reserve in ageing and Alzheimer’s disease. Lancet Neurol. 11, 1006–1012 (2012). 23079557 10.1016/S1474-4422(12)70191-6 PMC3507991

[35] National Guideline Centre. Hearing Loss in Adults: Assessment and Management. NICE Guideline NG98 (National Institute for Health and Care Excellence, 2018). 30011159

[36] NHS Business Services Authority. Sight tests, glasses and contact lenses. NHSBSA https://www.nhsbsa.nhs.uk/help-travel-eye-care-wigs-and-fabric-support-costs/sight-tests-glasses-and-contact-lenses (2024).

[37] Bernabei R. Multicomponent intervention to prevent mobility disability in frail older adults: randomised controlled trial (SPRINTT project). BMJ 377, e068788 (2022). 35545258 10.1136/bmj-2021-068788 PMC9092831

[38] Cameron I. D. A multifactorial interdisciplinary intervention reduces frailty in older people: randomized trial. BMC Med. 11, 65 (2013). 23497404 10.1186/1741-7015-11-65 PMC3751685

[39] Hoogendijk E. O. Frailty: implications for clinical practice and public health. Lancet 394, 1365–1375 (2019). 31609228 10.1016/S0140-6736(19)31786-6

[40] Dent E. Physical Frailty: ICFSR International Clinical Practice Guidelines for Identification and Management. J. Nutr. Health Aging 23, 771–787 (2019). 31641726 10.1007/s12603-019-1273-z PMC6800406

[41] Salemme S., Mukadam N., Bodryzlova Y. Operationalising dementia prevention as a measurable NCD priority. Lancet Public Health 11, e277–e278 (2026). 41895305 10.1016/S2468-2667(26)00028-9

[42] United Nations General Assembly. Political declaration of the fourth high-level meeting of the General Assembly on the prevention and control of non-communicable diseases and the promotion of mental health and well-being. UN Resolution adopted 15 December 2025.

[43] Searle S. D., Mitnitski A., Gahbauer E. A., Gill T. M. & Rockwood K. A standard procedure for creating a frailty index. BMC Geriatr. 8, 24 (2008). 18826625 10.1186/1471-2318-8-24 PMC2573877

[44] Tsimpida D., Kontopantelis E., Ashcroft D. & Panagioti M. Comparison of self-reported measures of hearing with an objective audiometric measure in adults in the English Longitudinal Study of Ageing. JAMA Netw. Open 3, e2015009 (2020). 32852555 10.1001/jamanetworkopen.2020.15009 PMC7453309

[45] Fan J., Yu C., Guo Y., Bian Z., Sun Z., Yang L., Chen Y., Du H., Li Z., Lei Y., Sun D., Clarke R., Chen J., Chen Z., Lv J., Li L. & China Kadoorie Biobank Collaborative Group. Frailty index and all-cause and cause-specific mortality in Chinese adults: a prospective cohort study. Lancet Public Health 5, e650–e660 (2020). 33271078 10.1016/S2468-2667(20)30113-4 PMC7708389

[46] Langa K. M., Larson E. B., Crimmins E. M., Faul J. D., Levine D. A., Kabeto M. U. & Weir D. R. A comparison of the prevalence of dementia in the United States in 2000 and 2012. JAMA Intern. Med. 177, 51–58 (2017). 27893041 10.1001/jamainternmed.2016.6807 PMC5195883

[47] Cadar D., Lassale C., Davies H., Llewellyn D. J., Batty G. D. & Steptoe A. Individual and area-based socioeconomic factors associated with dementia incidence in England: evidence from a 12-year follow-up in the English Longitudinal Study of Ageing. JAMA Psychiatry 75, 723–732 (2018). 29799983 10.1001/jamapsychiatry.2018.1012 PMC6145673

[48] Kim S., Myers L., Wyckoff J., Cherry K. E. & Jazwinski S. M. The frailty index outperforms DNA methylation age and its derivatives as an indicator of biological age. GeroScience 39, 83–92 (2017). 28299637 10.1007/s11357-017-9960-3 PMC5352589

[49] Zhou S., Chen G., Fong T.-L., Tang G., Xiong R., Sun Y. X., Lu J., Wang N. & Feng Y. Joint association of frailty index and biological aging with all-cause and cause-specific mortality: a population-based longitudinal cohort study. Arch. Gerontol. Geriatr. 134, 105856 (2025). 40228393 10.1016/j.archger.2025.105856

[50] Stolz E., Mayerl H., Hoogendijk E. O., Armstrong J. J., Roller-Wirnsberger R. & Freidl W. Acceleration of health deficit accumulation in late-life: evidence of terminal decline in frailty index 3 years before death in the US Health and Retirement Study. Ann. Epidemiol. 58, 156–161 (2021). 33812966 10.1016/j.annepidem.2021.03.008

[51] Yang Y. & Lee L. C. Dynamics and heterogeneity in the process of human frailty and aging: evidence from the U.S. older adult population. J. Gerontol. B Psychol. Sci. Soc. Sci. 65B, 246–255 (2010). 20007299 10.1093/geronb/gbp102 PMC2981448

[52] Marshall A., Nazroo J., Tampubolon G. & Vanhoutte B. Cohort differences in the levels and trajectories of frailty among older people in England. J. Epidemiol. Community Health 69, 316–321 (2015). 25646207 10.1136/jech-2014-204655 PMC4392235

[53] Song X., Mitnitski A. & Rockwood K. Prevalence and 10-year outcomes of frailty in older adults in relation to deficit accumulation. J. Am. Geriatr. Soc. 58, 681–687 (2010). 20345864 10.1111/j.1532-5415.2010.02764.x

[54] Fang M. Lifetime risk and projected burden of dementia. Nat. Med. 31, 772–776 (2025). 39806070 10.1038/s41591-024-03340-9 PMC12305800

[55] Wang J. Cross-national analysis of social determinants of frailty among middle-aged and older adults: a machine learning study in the USA, England, and China. Humanit. Soc. Sci. Commun. 12, 866 (2025).

[56] van Buuren S. & Groothuis-Oudshoorn K. mice: multivariate imputation by chained equations in R. J. Stat. Softw. 45, 1–67 (2011).

[57] Jackson C. H. Multi-state models for panel data: the msm package for R. J. Stat. Softw. 38, 1–28 (2011).

[58] van den Hout A. Multi-State Survival Models for Interval-Censored Data (Chapman & Hall/CRC, 2017).

[59] Putter H., Fiocco M. & Geskus R. B. Tutorial in biostatistics: competing risks and multi-state models. Stat. Med. 26, 2389–2430 (2007). 17031868 10.1002/sim.2712

[60] Sabia S., Dugravot A., Dartigues J. F., Abell J., Elbaz A., Kivimäki M. & Singh-Manoux A. Physical activity, cognitive decline, and risk of dementia: 28 year follow-up of Whitehall II cohort study. BMJ 357, j2709 (2017). 28642251 10.1136/bmj.j2709 PMC5480222

[61] Mansournia M. A. & Altman D. G. Population attributable fraction. BMJ 360, k757 (2018). 29472187 10.1136/bmj.k757

